# Feasibility and acceptability of electronic administration of patient reported outcomes using mHealth platform in emergency department patients with non-medical opioid use

**DOI:** 10.1186/s13722-021-00276-0

**Published:** 2021-11-10

**Authors:** Kathryn Hawk, Caitlin Malicki, Jeremiah Kinsman, Gail D’Onofrio, Andrew Taylor, Arjun Venkatesh

**Affiliations:** 1 Department of Emergency Medicine, Yale University School of Medicine, 464 Congress Ave, Suite 260, New Haven, CT 06519 USA; 2 Center for Outcomes Research, Yale School of Medicine, New Haven, CT 06519 USA; 3 Yale Program in Addiciton Medicine, Yale School of Medicine, New Haven, CT 06519 USA

**Keywords:** Opioid use disorder, Patient reported outcome measures, Mobile health, mHealth, Emergency department

## Abstract

**Background:**

The emergency department (ED) offers an important opportunity to identify patients with opioid use disorder (OUD) and initiate treatment. However, post-ED follow-up is challenging, and novel approaches to enhance care transitions are urgently needed. Outcomes following ED visits have traditionally focused on overdose, treatment engagement, and mortality with an absence of patient reported outcomes (PROs), for example patient ability to schedule follow-up OUD treatment appointments or pick up a prescription medication, that may better inform evaluation of treatment pathways and near-term outcomes after acute events. In the context of increasing novel secure mobile health (mHealth) platforms, we explored the feasibility and acceptability of electronically collecting PROs from ED patients with non-medical opioid use to enhance care in the ED and transitions of care.

**Methods:**

ED patients with non-medical opioid use or opioid overdose who endorsed willingness and ability to complete electronic surveys after discharge were enrolled from a tertiary, urban academic ED. Participants were enrolled in an mHealth platform, shared electronic health records with researchers, and completed electronic surveys of PROs at baseline, three- and thirty-days post discharge from the hospital, including questions about ability to schedule a follow-up appointment, pick up a prescription medication and overdose risk behaviors. Primary outcomes were measures of feasibility and acceptability of electronic PRO collection among ED patients with non-medical opioid use.

**Results:**

Among 1,808 patients assessed for eligibility between June-December 2019, 101 of 130 (78%) eligible adult patients consented to participate. Ninety-six (95%) of 101 patients completed registration in the mHealth platform, and 77/96 (80%) were successful in sharing their electronic health data. Completion rates for the baseline, three-day and thirty-day surveys were 97% (93/96), 49% (47/96) and 42% (40/96). Implementation challenges included short engagement window during ED visit, limited access to smartphones/computers, insufficient battery life of participant phone to access email and password, forgotten emails and passwords, multi-step verification processes for account set-up, and complaints about hospital care, most of which were effectively addressed by study personnel.

**Conclusions:**

ED patients with OUD were willing to share electronic health information and PROs, although implementation challenges were common, and more than half of participants were lost-to-follow-up after hospital discharge at 30 days. Efforts to streamline communication and remove barriers to engagement are needed to improve the collection of PROs and pathways of care in ED patients with OUD.

*Clinical Trial Registration* ClinicalTrials.gov (NCT03985163). Date of Registration: June 10, 2019, Retrospectively registered (First enrollment June 8, 2019). https://clinicaltrials.gov/ct2/show/record/NCT03985163

## Background

The US has seen rapidly increasing opioid-associated morbidity and mortality, with increased rates of fatal and non-fatal overdose and opioid-related utilization of inpatient and emergency department (ED) care [1–4]. ED visits for opioid-related adverse drug events, complications of injection drug use, and opioid withdrawal have become increasingly common, resulting in ED visits for opioid-related presentations more than doubling between 2010 and 2018 [5, 6]. After a brief decrease in opioid-associated deaths from 2017 to 2018, a 4.6% increase in drug overdose deaths (70,980) were reported in 2019, with 50,042 deaths attributed to opioids [7]. Alarmingly, this trend has continued, with provisional reporting from Centers for Disease Control and Prevention of more than 94,0000 deaths due to drug overdose deaths between January 2020 and January 2021, a 30.9% increase from the previous year [7]. A large treatment gap exists, with approximately 10% of individuals meeting criteria for a substance use disorder having received formal addiction treatment within the past year [8]. As EDs across the US provided care for almost 130 million annual ED visits in 2018, the ED offers a critical opportunity to identify and initiate treatment for patients with OUD, which has been shown to increase treatment engagement [9, 10]. However, effective linkages to outpatient care post-discharge from the ED can present challenges in real world settings. Novel approaches to support patients and enhance connection with outpatient treatment and resources that can be integrated into regular ED clinical practice are needed.

Mobile health (mHealth) technology, defined by the National Institutes for Health as “the use of mobile and wireless devices to improve health outcomes, healthcare services and health research,” has significant potential in enhancing follow up among patients with non-medical opioid use and OUD [11]. Additionally, the movement towards a more patient-centered healthcare system through the collection of patient reported outcomes (PROs) has been a priority for patients, providers and federal agencies [12–15]. PROs are data that come directly from the patient without any additional interpretation that provide clinically meaningful insight into screening, diagnosis, treatment response, functional outcomes or overall health status of a patient [12, 16]. While PROs have been used to evaluate many therapeutics and devices, their use to evaluate the effectiveness of treatment referral strategies and programs is in its infancy. We therefore performed a pilot study to test the feasibility and acceptability of electronically capturing PROs using mHealth technology among patients with non-medical opioid use in an ED setting. We hypothesized that collecting PROs using mHealth technology would be feasible and acceptable to ED patients with non-medical opioid use.

## Methods

### Study design and setting

We conducted an observational study of ED patients with non-medical opioid use. Participants were enrolled from the ED at Yale New Haven Hospital (YNHH), a 1,450-bed teaching hospital located in New Haven, Connecticut with a catchment area of 350,000 that includes a diverse ethnic and cultural mix; non-Latino white (48%), non-Latino Black (30%), Latino (18%), Other (2.5%) and Asian (< 1%) and is the most nationally representative community in terms of socioeconomics and education [17]. The YNHH ED has 58 treatment spaces and an estimated 106,600 annual patient encounters. The study received Institutional Review Board approval at Yale University and was registered at Clinicaltrials.gov (NCT03985163).

### Characteristics of participants

The study enrolled patients presenting to the YNHH ED for opioid overdose or those screening positive for OUD or non-medical opioid use, based on the NIDA Quick Screen, a brief 30-day substance use screener [18]. Eligible participants were identified by electronic health record (EHR), emergency clinician referral and bed-to-bed screening and were enrolled at the bedside between 6am and 11 pm daily. Exclusion criteria included age < 18 years, non-English speaking, active psychiatric evaluation, and inability to provide consent or follow-up contact information. Over the course of study recruitment, RAs developed a more effective process for enrolling patients who were cleared by the psychiatric team after evaluation prior to ED discharge.

### Study intervention

**Fig. 1:**
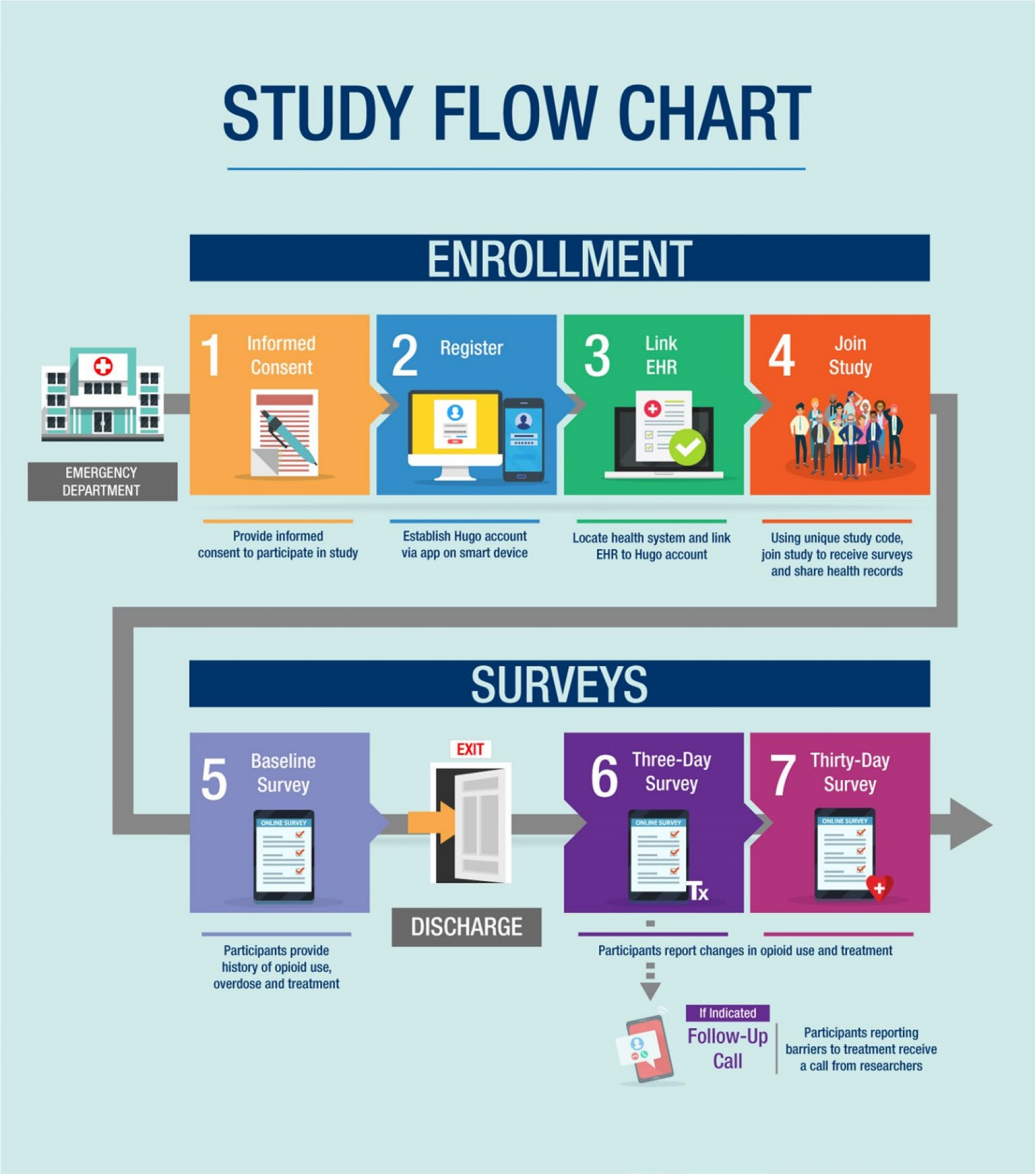
Study flow

After providing informed consent, participants enrolled in an mHealth platform, Hugo, and were asked to (1) access and share EHR data with the study and (2) complete electronic questionnaires at enrollment, 3-day and 30-days post discharge from the hospital accessible by an electronically delivered link by text or email. A detailed study flow is illustrated in Fig. 1.

#### mHealth platform

Hugo is a sync-for-science platform that allows patients to access personal data from multiple health systems, share data with researchers, and complete surveys for researchers using personal email and/or mobile devices [19]. To enroll in Hugo, participants were asked to access the app via a tablet or smartphone, create an account using a personal email, link available EHR data through their healthcare system’s secure online patient portal (“MyChart” at YNHH), and join the study to receive surveys. Participants agreed to share EHR data with researchers for one year following enrollment. As needed, a tablet and/or laptop were provided to participants during the registration process, and a research assistant (RA) assisted in addressing any challenges encountered such as password recovery, setting up and accessing a personal email, and creation of a MyChart account. If participants were admitted to the hospital during enrollment, the RA continued the registration process in the new patient location, as permitted, and follow-up surveys were scheduled following discharge from the hospital.

#### PRO administration

Based on patient preference, surveys were delivered by email and/or text using a secure link and could be completed on any computer or smart device. No questions were mandatory, and participants were permitted to skip questions they preferred not to answer. Surveys were considered “complete” if they had ≥ 1 answer, and participants clicked through the entire survey and received a gift card. Participants received a minimum of four email, text or phone reminders as needed and were given up to 30 days to complete each survey, after which they were considered lost to follow-up (LTFU). The baseline, 3-day and 30-day surveys included 12, 33–42 and 44–56 questions total, based on skip logic. Participants received a $10 electronic gift card for each survey completion, which was delivered automatically through a third-party vendor (Tremendous). Participants were given the option to receive an electronic gift card by text or email instantly or provide an address to have the gift card mailed. Electronic gift cards could be redeemed online only, while mailed gift cards were redeemable at brick and mortar stores.

#### Patient reported outcomes

PRO selection was guided by our interdisciplinary steering committee that included technical experts and representatives from National Institute on Drug Abuse, National Library of Medicine, Centers for Disease Control and Prevention, U.S. Food and Drug Administration, U.S. Department of Health and Human Services, academic researchers, and a community member with personal experience with OUD. Survey questions represent a mix of investigator generated questions designed to collect outcomes that could inform ED quality improvement for which validated surveys were not available (i.e. difficulty scheduling a follow-up appointment or filling a medication) and validated structured instruments designed to collect PROs (PROMIS surveys). The baseline survey consisted of demographics, DSM-5 criteria for OUD, and history of overdose and treatment for OUD, while the 3-day and 30-day surveys included recent treatment and prescription history, an overdose risk behavior survey as well as standardized patient reported outcomes including PROMIS Global-10, PROMIS Severity of Substance Use (Past 30 days) and Treatment Effectiveness Assessment (TEA) [20]. The PROMIS Global-10 includes questions on the general domains of health and functioning including overall physical health, mental health, social health, pain, fatigue, and overall perceived quality of life [21]. The PROMIS Severity of Substance Use includes questions about drug use other than alcohol or prescribed medications in past 30 days [22, 23].

#### Follow-up call

As EDs often have staff responsible for making follow-up patient calls regarding late resulting tests (blood and urine cultures, results finalized after patient discharge) referrals, or patient satisfaction, this step was designed to test the feasibility of a pragmatic strategy to integrate PRO feedback to enhance patient outcomes using an existing resource at many EDs. Following the 3-day questionnaire only, a phone call was triggered if the patient reported challenges picking up a prescription or scheduling a follow-up appointment for OUD treatment. During the phone call, a trained RA provided information and resources about OUD treatment, as appropriate. Challenges were captured qualitatively based on interviews and feedback from study RAs.

#### Feasibility and acceptability outcomes

Our feasibility outcomes included the proportion of eligible patients who were willing to enroll in the study overall and the ability to link patient’s EHR to the mHealth platform. Acceptability outcomes include response rates to the baseline, 3-day and 30-day surveys and the ability to reach patients by telephone when a PRO triggered a follow-up telephone call.

### Data collection and analysis

Screening, enrollment, and follow-up data were recorded by study RAs in case report forms using the Qualtrics XM Platform. Survey data were collected and stored by Hugo and were extracted and transferred via the Hugo platform by researchers. At the end of the study, Hugo staff shared a comprehensive analytic dataset including EHR data for all participants via secure transfer. Using a unique identifier to link participants, all data were merged in Microsoft Excel and analysis was performed using SAS version 9.4. For analysis purposes, “completers” included participants completing two or more surveys and “non-completers” completed no surveys or baseline only, as nearly all baseline surveys (97%) were completed during enrollment in the ED and do not accurately capture engagement post-discharge from the hospital. As a pilot feasibility study, no power analyses were used.

## Results

### Study participants and feasibility outcomes

**Fig. 2:**
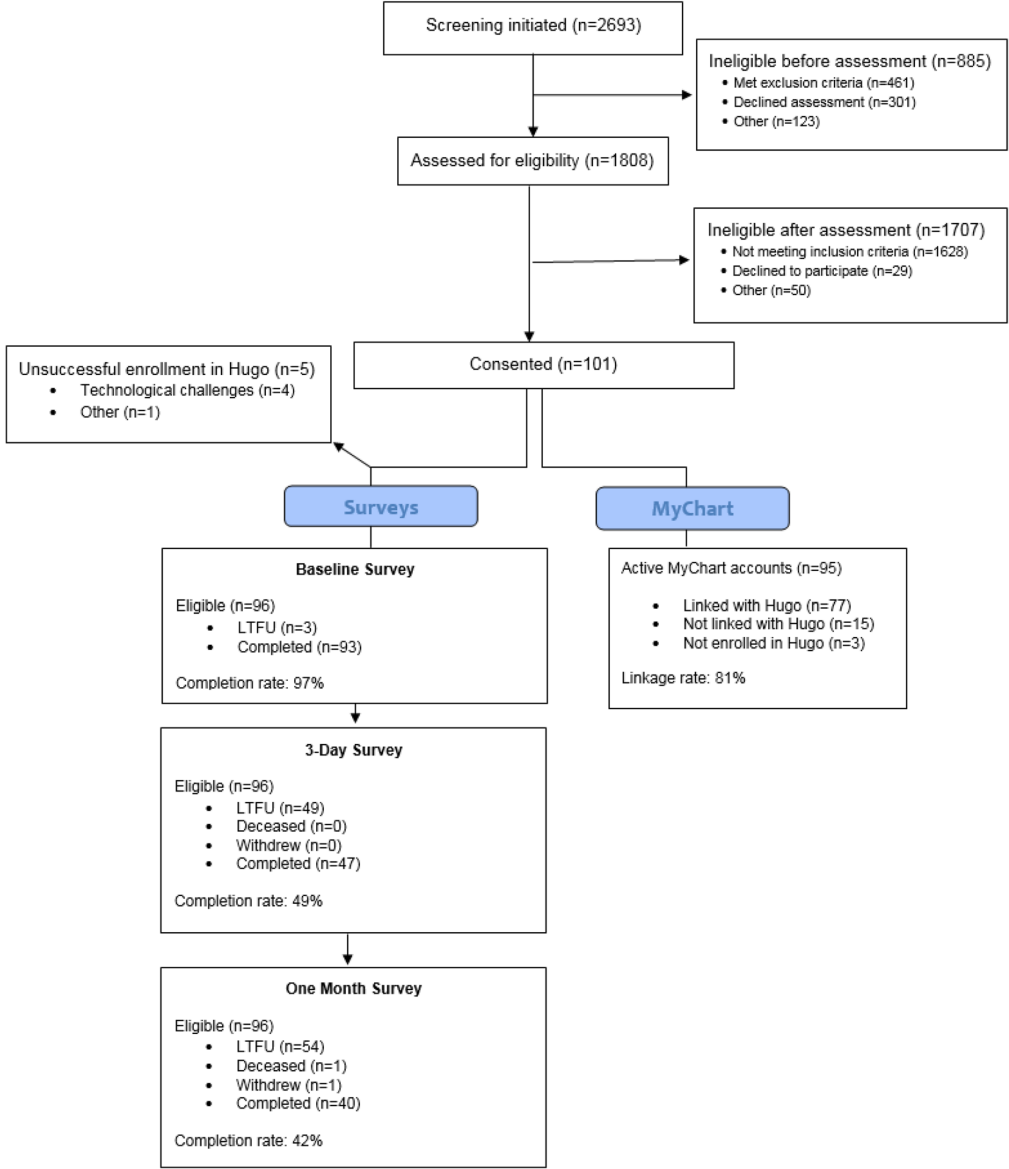
CONSORT

As depicted in the CONSORT diagram (Fig. 2), 1,808 patients were assessed for eligibility between June 7 and December 13, 2019, and 101 of 130 (78%) eligible adult patients with non-medical opioid use, OUD or opioid overdose consented to participate. Characteristics of enrolled participants are described in Table 1. Among eligible patients, reasons for nonparticipation included not interested in research (n = 20), time constraints (n = 3), data privacy concerns (n = 2) and other (n = 4) (Table 2). During the follow-up period, 1 participant withdrew, 1 died and 22 had non-working or disconnected telephone numbers when contacted for the three-day and/or thirty-day survey reminders.

**Table 1:** Characteristics of Participants by Survey Completion

	All (N = 101)	Non-completers (0–1 surveys completed)* (N = 53)	Completers (2–3 surveys completed*) (N = 48)
Sex (male)	52 (51.49%)	28 (52.83%)	21 (43.75%)
Age (years; mean ± SD)	38.41 (10.25)	40.28 (11.61)	36.35 (8.14)
Race			
White	76 (75.25%)	39 (73.58%)	37 (77.08%)
Black	16 (15.84%)	8 (15.09%)	8 (16.67%)
Other	9 (8.91%)	6 (11.32%)	3 (6.25%)
Ethnicity			
Hispanic	9 (8.91%)	5 (9.43%)	4 (8.33%)
Non-Hispanic	92 (91.09%)	48 (90.57%)	44 (91.67%)
Insurance			
Medicaid	88 (87.13%)	45 (84.91%)	43 (89.58%)
Medicare	5 (4.95%)	2 (3.77%)	3 (6.25%)
Private	7 (6.93%)	5 (9.43%)	2 (4.17%)
Uninsured	1 (0.99%)	1 (1.89%)	0 (0.00%)
OUD Severity (DSM-5)^†^			
None	4 (4.44%)	2 (4.65%)	2 (4.26%)
Mild	4 (4.44%)	2 (4.65%)	2 (4.26%)
Moderate	6 (6.67%)	1 (2.33%)	5 (10.64%)
Severe	76 (84.44%)	38 (88.37%)	38 (80.85%)

^*^ “Non-completers” completed zero surveys or baseline only; “completers” completed two or more surveys.

^†^OUD Severity collected from baseline survey available to the 96/101 participants that successfully registered with Hugo, with 90 responses provided

**Table 2:** Patient reasons for non-participation

	n	Percent
Ineligible before assessment (n = 885)		
Met exclusion criteria		
Inability to provide consent	250	28
Limited English proficiency	160	18
< 18 years old	15	2
Declined assessment	301	34
Other		
Police custody	48	5
Admitted and not followed	45	5
Awaiting emergent psychiatric evaluation	36	4
Medically unstable	13	1
Enrolled in other research	9	1
Left AMA	8	1
Ineligible after assessment (n = 1707)		
Met exclusion criteria		
Unable to consent	10	1
Active emergency psychiatry patient	1	0
Not meeting inclusion criteria		
Opioid inclusion not met	1585	93
Unwilling to complete surveys	24	1
Unable to complete surveys	19	1
Declined to participate		
Not interested in research	20	1
Data privacy concerns	2	0
Time constraints	3	0
Declined for other reasons	4	0
Other	39	2

Ninety-six out of 101 patients registered an account with Hugo, of which 80% (n = 77) shared electronic health data. Among patients registered with Hugo, 81 opted to receive surveys by email only, and 15 opted to receive surveys by email and text. Reasons for failed Hugo registration included technological challenges (link not sending despite troubleshooting, etc.) (n = 4) and time constraints (n = 1). Reasons for failed EHR linkage among 15 Hugo users included challenges activating an account (potentially user error or connectivity issues), difficulty accessing existing account (account locks after three failed login attempts), and trouble linking MyChart with Hugo (password or connectivity issues with Hugo). Forty-four (44%) patients needed help establishing an email account prior to registering with Hugo, 35 (35%) needed help accessing their existing email account, 77 (77%) patients needed assistance creating an account in the online EHR portal, MyChart, and 14 (14%) needed help accessing their existing MyChart account. Compared to participants who did not require assistance creating an email account, those who required assistance were significantly less likely to complete the 3-day and 30-day follow-up survey (see Table 3). Additional challenges encountered during enrollment included integrating enrollment with clinical care, particularly when patients were uncomfortable or had needs unattended to by ED staff, Wi-Fi connectivity issues, and limited access to personal telephones based on departmental protocols driven by patient physical location rather than clinical state and a scarcity of phone chargers and outlets. RA feedback and patient communications indicated an overall willingness to share EHR data.

**Table 3:** Three- and thirty-day survey completion by whether ra assistance was required to create an e-mail account

Needed help creating email account	Not completed	Completed	Total	Chi-square p-value
3-day follow-up survey completion				
No	11 (22.92%)	37 (77.08%)	48	< .0001
Yes	35 (79.55%)	9 (20.45%)	44	
Total	46	46	92	
30-day follow-up survey completion				
No	16 (33.33)	32 (66.67)	48	< .0001
Yes	38 (86.36)	6 (13.64)	44	
Total	46	46	92*	

*Data on whether assistance was required in creating an e-mail account were missing for 4 of the 96 participants who successfully enrolled in Hugo

### Acceptability outcomes

Completion rates for the baseline, three-day and thirty-day surveys were 97% (93/96), 49% (47/96) and 42% (40/96), and details about survey completion are described in Table 4. In brief, among 47 participants completing the three-day survey, responses from 14 (30%) triggered a follow-up telephone call based on identified barriers to obtaining a substance use treatment appointment, of which five participants (36%) were successfully reached. Ninety-one (95%) participants received at least one automated survey reminder for the three-day and thirty-day survey, which were completed post-discharge. The mean time from survey distribution to completion was 5.3 days. A total of 6 participants started but discontinued the survey, answering a mean of 8 (13 SD) questions before abandoning. Among all 180 surveys completed throughout this study, 67 (37%) had all questions complete, and 113 (63%) surveys had at least one unanswered question. The median number of skipped questions for each survey was 0, 5 and 5.5 for the baseline, three-day and thirty-day surveys, respectively. Among survey completers, participants spent a median of 2 min, 6 min and 5.5 min on the baseline, three-day and thirty-day survey, respectively. Among follow-up surveys completed outside of the hospital (n = 87), 6% were completed in the morning (5am–12 pm); 26% in the afternoon (12 pm–4 pm); 28% in the evening (4–8 pm) and 40% were completed overnight (8 pm–5am). There were no adverse events, although two events from two different participants were shared with the IRB that involved the communication of sensitive information and complaints about their care in the ED, both of which were unrelated to the study and unrelated to data collected on study assessments.

**Table 4:** Summary of survey characteristics and completion rates

	Baseline survey N = 93	Three-day survey N = 47	Thirty-day survey N = 40	Total N = 180
Total questions	12	33–42	44–56	101
Time from survey distribution to completion (mean)	N/A	6.2 days	4.1 days	5.3 days
Started and abandoned surveys (n)	0 (0%)	3 (6%)	3 (7.5%)	6 (3%)
Surveys with ≥ 1 skipped question	29 (31%)	45 (96%)	39 (97.5%)	113 (63%)
Skipped questions (median)	0	5.0	5.5	11.0
Time spent on survey (median)	2 min	6 min	5.5 min	17.5 min
Time of day completed				
Morning (5 am–12 pm)	NA	2 (4.2%)	3 (7.5%)	(5.8%)
Afternoon (12–4 pm)	NA	11 (23.4%)	12 (30.0%)	(26.4%)
Evening (4–8 pm)	NA	16 (34.0%)	8 (20.0%)	(27.6%)
Overnight (8 pm–5 am)	NA	18 (38.3%)	17 (42.5%)	(40.2%)

## Discussion

In this pilot study utilizing a novel mHealth technology within the unique ED setting, we found that patients with non-medical opioid use were willing to share electronic health information and provide PRO data related to non-medical opioid use and the navigation of post-ED care. We found that collecting PROs through a mHealth platform was feasible and acceptable to ED patients with non-medical opioid use. Several key implementation challenges were identified, but notably, despite public and media attention to data security, patient concern about linking electronic health data across platforms was not a prominent challenge or noted limitation. Patients were willing to use mHealth technology to provide PROs relating to details of ED visit and SUD referral, ability to pick up prescribed medications, ability to schedule follow-up appointments and additional measures relating to quality of life and substance use. Additionally, ED patients with non-medical opioid use may be more amenable to providing PRO data related to ED care and to identify assistance needed with transitions of care when collected through a mHealth platform due to reduced stigma. Therefore, collection of PROs through an mHealth platform in ED patients with non-medical opioid use offers a potential strategy to gather patient-focused data and to inform a feedback loop to help facilitate assistance with prescription medications or follow-up appointments that would be feasible within usual ED clinical workflows and outside of a research setting.

Overall, 95% of patients were able to complete their registration in the mHealth platform, and 80% were successful in sharing their electronic health data. Several challenges were identified within this pilot, including logistical, technical and patient-specific barriers, most of which have been identified in other non ED-based mHealth PRO surveys [24]. We identified several challenges consistent with prior pilot studies of mHealth platforms, including difficulty retaining email, mHealth and EHR login information, and integrating enrollment into clinical care; technical issues included challenges with Wi-Fi connectivity, delays in data uploading; and patient specific factors included overall comfort and management of clinical symptoms [24, 25]. Many of these barriers were addressable by training of research staff, reiterating real-time communication with our IT and mHealth program support when needed and by consideration of patient comfort when enrolling a patient, with attention to unmet needs (blanket, food, relaying unmet needs to clinical team, etc.). Other strategies to overcome barriers included the purchase of multiple phone chargers for both android and iPhones to facilitate the recovery of e-mail passwords and training RAs to assist patients in the generation of new patient e-mail accounts. Given significant variation in follow-up survey completion for participants who received assistance with either setting up an email account or password recovery, additional strategies outside of providing a paper for them to record login information should be considered to enhance ability to participants to retain email login and password and attention to patient preference for telephone, text or e-mail contact should be considered for future studies and ED based interventions in this population.

Importantly, lack of access to telephone for the vast majority of ED patients with non-medical opioid use was not a barrier to participation, which is consistent with a prior study finding that an estimated 95% of patients in our ED report having cell phone access, and that smartphone access remains high among patients with substance use disorders [26]. Consistent with our and others experience, the existence of a telephone number on enrollment does not always translate to the ability to reach a participant for follow-up, likely due to a variety of participant and phone-specific problems including disconnected telephone numbers, absence of minutes, the use of transient or burner cellular phones and lost or stolen cellular phones. Prior research done in our ED evaluated variations in the ability to reach low-income smokers by time of month, finding that study participants were less likely to be reachable by telephone during the last week of the month compared to earlier weeks, which may reflect variations associated with monthly paychecks or other factors [27]. One strategy for minimizing loss to follow-up would be to test participant phone numbers in the ED using a study phone to minimize transcription errors and intentionally or inadvertently being given wrong telephone numbers. Additionally, based on initial challenges around enrollment being limited to ED patients not requiring acute psychiatric evaluation or admission, our RAs were able to develop a more effective process for enrolling patients who were cleared by the psychiatric team after evaluation prior to ED discharge to maximize engagement of ED patients who had been evaluated and cleared by psychiatry. Although we did encounter barriers, we found that our overall enrollment strategy to use an mHealth platform to link EHR data and deliver PRO surveys was feasible in this population of ED patients with non-medical opioid use.

Completion rates for the baseline, three-day and thirty-day surveys in our study were 97%, 49%, and 42% respectively. While the three-day and thirty-day response rates are low, they are not entirely inconsistent with other ED and hospital-based follow-up surveys. One survey of patients admitted with acute traumatic injury found that 19/26 participants completed the survey with a three month follow-up rate of 38% [24]. Another study evaluating the differences in follow-up participation based on automated survey data collection versus a more labor intensive, high-intensity collection of PROs surrounding more than 5,700 orthopedic surgeries reported a pre-procedure survey completion rate of 86% vs 100%, 3- or 6-month post-operative response rate of 55% vs 84% and 12-month follow-up of 53% vs 83% based on low- versus high-intensity data collection, respectively [25]. Our follow-up reminders included only 4 attempts by either phone call and/or text in order to more closely match usual ED clinical follow-up policies, which is well-suited to generalizing the use of this approach outside of research settings but is far fewer follow-up attempts than typically used to maximize follow-up for traditional clinical research trials [28, 29]

In future studies follow-up rates may be enhanced without significantly increasing follow-up attempts by “testing” participant follow-up phone numbers in the ED, collecting participants preferred time of day for follow-up calls and by collecting patients preferred social media method of contact if applicable (e.g. Facebook). Beyond the frequency of follow-up attempts, prior work has suggested that multi-modal approaches to follow-up may be more successful and implementation of PRO collection within this study may have also resulted in greater follow-up at the risk of limiting generalizability given our goal of evaluating feasibility of integration into a standard clinical workflow [30].

There are several limitations associated with this study. Participant concerns around the feasibility and acceptability of sharing EHR and PROs may be mitigated by the presence of in-person RAs or by incentive for study compensation. Individuals who were willing to participate in the study may not be generalizable to all ED patients with non-medical opioid use. Additionally, the development of an effective collaboration with our emergency psychiatry colleagues designed to enhance enrollment of ED patients who had been evaluated and cleared by psychiatry prior to discharge led to an overrepresentation of patients who were enrolled after a psychiatric evaluation, as patients were in the ED longer and therefore easier to capture for enrollment.

## Conclusions

ED patients with OUD were willing to share electronic health information and PROs, although implementation challenges were common, and about half of participants were lost-to-follow-up within 30 days of hospital discharge within a real-world, mHealth application with modest follow-up effort. Future work to streamline integration and remove barriers to engagement is needed to improve the collection of PROs and to advance patient centered care initiatives for patients with OUD.

## Data Availability

All data generated or analyzed during this study are included in this published article.

## Abbreviations

ED: Emergency department
IRB: Intuitional Review Board
mHealth: Mobile health
OUD: Opioid use disorder
PRO: Patient reported outcome
RA: Research assistant
TEA: Treatment Effectiveness Assessment
YNHH: Yale New Haven Hospital
